# Lumbar posterior group muscle degeneration: Influencing factors of adjacent vertebral body re-fracture after percutaneous vertebroplasty

**DOI:** 10.3389/fmed.2022.1078403

**Published:** 2023-04-17

**Authors:** Ming Chen, Cekai Yang, Zhuoyan Cai, Youtao Liu, Hao Liu, Jianchao Cui, Zhensong Yao, Yuan Chen

**Affiliations:** ^1^The First Clinical Medical College, Guangzhou University of Chinese Medicine, Guangzhou, China; ^2^Department of Spinal Surgery, The First Affiliated Hospital of Guangzhou University of Chinese Medicine, Guangzhou, China; ^3^Department of Orthopaedics, Baiyun Hospital of the First Affiliated Hospital of Guangzhou University of Chinese Medicine, Guangzhou, China; ^4^College of Medicine and Recreation, Jiangyang City Construction College, Luzhou, China

**Keywords:** osteoporotic vertebral compression fracture, percutaneous vertebroplasty (PVP), vertebral body re-fracture, muscular degeneration of posterior group of lumbar spine, chronic diseases in the elderly

## Abstract

**Objective:**

The purpose of the study was to explore the influencing factors of adjacent vertebral re-fracture after percutaneous vertebroplasty (PVP) for osteoporosis vertebral compression fractures (OVCFs).

**Methods:**

We retrospectively analyzed the clinical data of 55 patients with adjacent vertebral re-fracture after PVP operation for OVCFs in our hospital from January 2016 to June 2019, they were followed up for 1 year and included in the fracture group. According to the same inclusion and exclusion criteria, we collected the clinical data of 55 patients with OVCFs without adjacent vertebral re-fracture after PVP in the same period and included them in the non-fracture group. We performed univariate and multivariate logistic regression analysis on the influencing factors of adjacent vertebral re-fracture in patients with OVCFs after PVP.

**Results:**

There were significant differences in body mass index (BMI), bone mineral density (BMD) *T*-value, amount of bone cement injected, bone cement leakage, history of glucocorticoid use, cross-sectional area (CSA), cross-sectional area asymmetry (CSAA), fat infiltration rate (FIR), and fat infiltration rate asymmetry (FIRA) of lumbar posterior group muscles [multifidus (MF) and erector spinae (ES)] between the two groups (*p* < 0.05). There was no significant difference in sex, age, or time from the first fracture to operation, the CAS, CSAA, FIR, and FIRA of psoas major (PS) between the two groups (*p* > 0.05). Multivariate logistic regression showed that a higher dose of bone cement, greater CSAA and FIR of multifidus, and higher CSAA of erector spinae were independent risk factors for recurrent fractures of adjacent vertebrae after PVP.

**Conclusion:**

There are many risk factors for recurrent vertebral fracture after PVP in patients with OVCFs, and degeneration of paraspinal muscles (especially posterior lumbar muscles) may be one of the risks.

## Introduction

In recent years, with the rapid development of the economy and society, the aging of the population has gradually intensified, and the problems of chronic diseases in the elderly are increasing day by day, especially chronic skeletal and muscle-related diseases. Osteoporosis is a systemic bone disease, which is prone to fracture due to the decrease of bone mass, the destruction of bone microstructure, and the increase of bone fragility (1). According to the “Compendium of Osteoporosis” released by the International Osteoporosis Foundation in 2015, the number of people at high risk of osteoporotic fracture worldwide was about 158 million in 2010 and will double by 2040 (2). Osteoporosis will bring great pressure on the social economy and medical care. As a common method for the treatment of OVCFs, the PVP has been recognized by clinicians and patients because of its high safety and efficacy.

However, clinical observation found that the overall curative effect was affected by the re-fracture of the adjacent vertebral body after PVP. Related clinical studies (3) have shown that many factors lead to a high incidence of adjacent vertebral body re-fracture after PVP. As the most active and load-bearing vertebral body, the lumbar vertebra plays an important role in the stability of the spine. The stability of lumbar vertebrae requires the joint action of vertebrae, muscles, intervertebral facet joints, connective tissue, nerves, and so on. The paraspinal muscle is mainly composed of the PS of the anterior group and the MF and ES of the posterior group. At present, there are few academic studies on the effect of paraspinal muscle degeneration on PVP. As deep muscles of the spine, the MF and ES are important factors to maintain the stability and normal physiological function of the low back spine. The decline of the mass of the MF and ES will accelerate spinal function degeneration.

Therefore, the purpose of this study was to explore the effect of paraspinal muscle degeneration on vertebral body re-fracture after PVP by observing the cross-sectional area and fat distribution of paravertebral muscles (multifidus, erector spinae, and psoas major) before the operation, so as to provide a new idea for the clinical prevention and treatment of adjacent vertebral body re-fracture after PVP.

## Research materials and methods

### Participants

We retrospectively analyzed the clinical data of 55 patients with adjacent vertebral re-fracture after PVP operation for OVCFs in the Department of Spinal Orthopedics, the First Affiliated Hospital of Guangzhou University of Chinese Medicine from January 2016 to June 2019 and included them in the fracture group. In addition, we collected the clinical data of 55 patients with OVCFs who did not have adjacent vertebral body re-fracture after PVP in the same period and were included in the non-fracture group. The inclusion criteria are as follows: Diagnosed as osteoporotic vertebral compression fracture and single compression fracture of thoracic or lumbar vertebrae (T1-L5); magnetic resonance imaging (MRI) examination of lumbar vertebrae was performed in our hospital before the first operation, and there were complete and clear lumbar MRI images. The patients were followed up for 1 year after PVP treatment. The patient knows and signs the consent form. Exclusion criteria were as follows: Did not pass PVP operation treatment, history of spinal internal fixation, complicated with severe infection (such as bacteremia), complicated with neoplastic diseases such as spinal tumor and liver cancer, history of severe mental illness, follow-up less than 1 year. Standard of adjacent vertebral body re-fracture: Complete or partial fracture of bone structure in adjacent segments of the original fracture site.

### General information

We collected the eligible patients’ age, sex, BMI, BMD, use of corticosteroids (oral or intravenous), bone cement infusion, cement leakage after the first operation, and time from the first fracture to operation (Table 1).

**TABLE 1:** Clinical baseline differences among participants.

		Non-fracture group	Fracture group	T/Z/X^2^	*P*-value
Gender	Male	7 (6.36%)	132 (11.82%)	2.20	0.14
	Female	48 (43.64%)	42 (38.18%)		
Age (years)		74.22 ± 9.61	76.84 ± 7.70	-1.58	0.12
BMI (kg/m^2^)		23.63 ± 4.91	21.35 ± 4.17	2.62	0.01
	<18.5	7 (6.36%)	11 (10%)		
	18.5–24.9	29 (26.36%)	41 (37.27%)		
	25.0–29.9	14 (12.73%)	2 (1.82%)		
	>29.9	5 (4.55%)	1 (0.91%)		
BMD (*T*-value)		−3.54 ± 1.22	−4.25 ± 1.09	3.40	<0.01
Time from the first fracture to the operation (days)		5.29 ± 1.81	5.71 ± 1.89	-1.18	0.24
Bone cement injection volume (ml)		4.8 (3.50–5.30)	5.40 (4.10–5.50)	-3.60	<0.001
Bone cement leakage	Yes	2 (1.82%)	8 (7.27%)	3.96	0.047
	No	53 (48.18%)	47 (42.73%)		
Use of glucocorticoids	Yes	5 (4.55%)	21 (19.09%)	12.89	<0.001
	No	50 (45.45%)	34 (30.91%)		

BMI, body mass index. A little thin (7), <18.5 kg/m^2^; Normal, 18.5–24.9 kg/m^2^; A little fat, 25.0–29.9 kg/m^2^; obesity, >29.9 kg/m^2^. BMD, bone mineral density.

### Magnetic resonance imaging data acquisition

All MRI data were obtained using a 1.5T MRI scanner (Magnetom avanto, Siemens; T1WI:TR700 ms, TE:12 ms; T2WI:TR5000 ms, TE:112 ms; slice thickness:4 mm) in our hospital. The patients took a supine position and underwent routine lumbar MRI examination to obtain a T1-weighted image (T1WI) and T2-weighted image (T2WI) of the sagittal position of lumbar vertebrae, then located the middle of L3/4 intervertebral disc by sagittal T2WI, and amputated its transverse axial T2WI. All the data were imported into ImageJ software and measured by two experienced orthopedic surgeons at the same time; when the results are inconsistent, it is up to them to decide after discussion.

### Measurement of related data of paraspinal muscle

**FIGURE 1:**
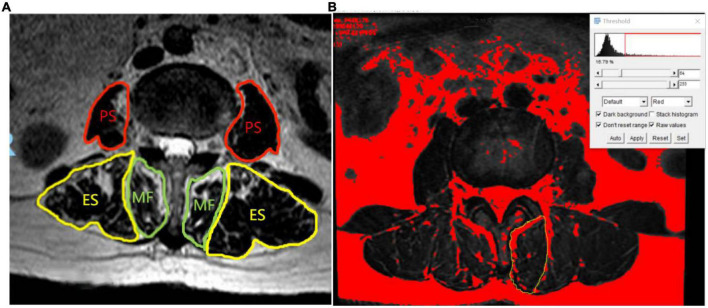
Measurement of cross-sectional area and intermuscular fat area of paraspinal muscle **(A)** (MF, multifidus; ES, erector spinae; PS, psoas major). **(B)** The automatic threshold setting of ImageJ distinguish fat from muscle. The range of the yellow curve is the left MF, and the red part is the fat area of MF.

Some studies have shown that (4) ImageJ software (version 1.5) has been widely used to measure paraspinal muscle CSA and FIR. The two physicians measured the related parameters of paraspinal muscles (MF, ES, and PS) on both sides of L3/4 segments. The specific measurement method is as follows: Import the target image into ImageJ and then set the scale, and the two doctors manually outline the paraspinal muscles on both sides along the deep fascia at the edge of the muscle the fascia and fat next to the spinous process are delineated into the MF-CSA. The fat between the multifidus muscle and the erector spine muscle is classified as the ES-CSA (5), and then, the fat area of the paraspinal muscles on both sides is measured by the automatic threshold function of the software (Figure 1).

The calculation method of paraspinal muscle parameters is as follows: The paraspinal muscles (MF, ES, and PS) of cross-sectional area (CSA): Sum of the CSA of left and right side of paraspinal muscles; cross-sectional area asymmetry (CSAA) of paraspinal muscles (5): (the CSA of paraspinal muscles on the larger side minus the CSA of paraspinal muscles on the smaller side)/the CSA of paraspinal muscles on the larger side, multiplied by 100%; fat infiltration rate (FIR) of paraspinal muscles (6): Fat area of paraspinal muscle divided by CSA of paraspinal muscles, multiplied by 100%; fat infiltration rate asymmetry (FIRA) of paraspinal muscles: (the FIR of paraspinal muscles on the larger side minus the FIR of paraspinal muscles on the smaller side)/the FIR of paraspinal muscles on the larger side, then multiplied by 100%.

### Data analysis

We use SPSS25.0 to analyze all the research data. All the measurement data first draw a histogram to determine whether it meets the normality. The measurement data that conform to normality are expressed as (average ± standard deviation), while those that do not conform to normality are described by median (IQR), and the counting data are represented by n (%). The measurement data in accordance with normality were analyzed by the independent sample *t*-test, the data of skewness distribution were analyzed by rank sum test (Wilcoxon test), and the counting data were analyzed by chi-square test. We further included the statistically significant data in binary logistic regression analysis to obtain independent risk factors for adjacent vertebral re-fracture after PVP.

## Results

### Clinical baseline characteristics of participants

The average age of all participants was 75.53 ± 8.77 (74.22 ± 9.61 in the non-fracture group and 76.84 ± 7.70 in the fracture group). Patients with lower BMI, lower BMD T-value, larger amount of bone cement, leakage of bone cement after PVP, and glucocorticoid use had a higher risk of recurrent fracture of adjacent vertebrae after PVP (all *p* < 0.05). There was no significant difference in sex, age, and the time from the first fracture to the operation between the two groups (all *p* > 0.05) (Table 1).

### Differences between groups of paraspinal muscles

**FIGURE 2:**
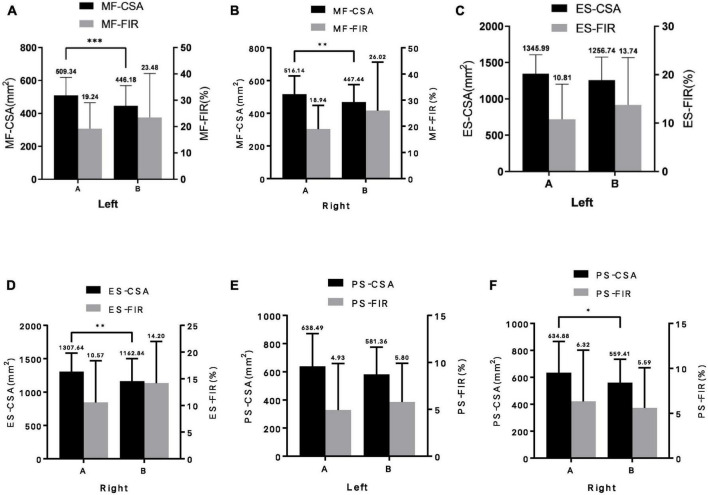
Difference of paraspinal muscles parameters between the two groups (A, non-fracture group; B, fracture group). **(A)** left multifidus; **(B)** right multifidus; **(C)** left erector spinae; **(D)** right erector spinae; **(E)** left psoas major; and **(F)** right psoas major. MF-CSA, cross-sectional area of multifidus; MF-FIR, fat infiltration rate of multifidus; ES-CSA, cross-sectional area of erector spinae; ES-FIR, fat infiltration rate of erector spinae; PS-CSA, cross-sectional area of psoas major; PS-FIR, fat infilt ration rate of psoas major; Left, left paraspinal muscle; Right, right paraspinal muscle. Data are reported as mean ± standard deviation of mean. *p < 0.05, ^**^p < 0.01, and ^***^p < 0.001.

The CSA of posterior spinal muscles (multifidus and erector spinae) in the non-fracture group was higher than that in the fracture group, but the CSAA, FIR, and FIRA of the posterior group muscles in the non-fracture group were lower than those in the fracture group (all *p* < 0.05). There was no significant difference in the CSA, CSAA, FIR, and FIRA of psoas major muscles between the two groups (all *p* > 0.05) (Table 2 and Figure 2).

**TABLE 2:** Difference of paraspinal muscles parameters between the two groups.

		Non-fracture group	Fracture group	T/Z	*P*-value
MF	CSA (mm^2^)	1025.48 ± 219.69	913.61 ± 218.31	2.68	<0.01
	CSAA (%)	5.39 (3.21–7.51)	14.77 (10.90–18.95)	-7.59	<0.001
	FIR (%)	19.09 ± 9.02	24.52 ± 15.42	-2.25	0.03
	FIRA (%)	19.52 (12.34–34.52)	29.68 (18.09–42.34)	-2.75	<0.01
ES	CSA (mm^2^)	2653.63 ± 533.09	2419.59 ± 628.87	2.11	0.04
	CSAA (%)	5.30 (2.78–7.72)	12.57 (9.35–19.78)	-6.82	<0.001
	FIR (%)	10.68 ± 7.24	13.87 ± 7.82	-2.22	0.03
	FIRA (%)	23.15 (15.72–43.63)	34.09 (21.95–48.27)	-2.42	0.02
PS	CSA (mm^2^)	1273.37 ± 445.16	1140.78 ± 352.58	1.73	0.09
	CSAA (%)	13.46 (6.59–24.23)	12.12 (5.75–22.28)	-0.49	0.62
	FIR (%)	4.32 (2.20–7.68)	4.97 (3.13–7.69)	-0.75	0.45
	FIRA (%)	46.60 (21.36–65.90)	44.89 (25.81–61.69)	-0.02	0.98

MF, multifidus; ES, erector spinae; PS, psoas major; CSA, cross-sectional area; CSAA, cross-sectional area asymmetry; FIR, fat infiltration rate; FIRA, fat infiltration rate asymmetry.

### Independent risk factors of re-fracture in patients with OVCFs

Multivariate logistic regression showed that increased amount of bone cement injection, the CSAA and FIRA of multifidus, and the CSAA of erector spinae were independent risk factors for recurrent adjacent vertebral fracture in patients with OVCFs after PVP treatment (Table 3).

**TABLE 3:** Multivariate logistic regression analysis of recurrent fracture after PVP.

Factors		β	Wald X^2^	OR	95% CI	*P*-value
BMI (Kg/m^2^)		-0.26	2.01	0.77	0.54–1.10	0.16
BMD (*T*-value)		-0.45	0.59	0.64	0.20–2.01	0.44
Bone cement injection volume (ml)		3.16	6.51	23.54	2.08–266.64	0.01
Bone cement leakage		3.76	1.32	42.92	0.07–26476.23	0.25
Use of glucocorticoids		-4.87	2.51	0.01	0.001–3.17	0.11
MF	CSA (mm^2^)	-0.001	0.05	1.00	0.99–1.01	0.83
	CSAA (%)	0.62	8.79	1.85	1.23–2.79	<0.01
	FIR (%)	-0.07	1.09	0.94	0.83–1.06	0.30
	FIRA (%)	0.11	3.99	1.12	1.002–1.25	0.046
ES	CSA (mm^2^)	0.003	2.63	1.003	1.00–1.01	0.11
	CSAA (%)	0.62	10.01	1.85	1.26–2.71	<0.01
	FIR (%)	0.12	0.87	1.13	0.87–1.46	0.35
	FIRA (%)	-0.09	2.61	0.91	0.82–1.02	0.11

BMI, body mass index; BMD, bone mineral density; MF, multifidus; ES, erector spinae; PS, psoas major; CSA, cross-sectional area; CSAA, cross-sectional area asymmetry; FIR, fat infiltration rate; FIRA, fat infiltration rate asymmetry.

## Discussion

Osteoporosis is a huge public health problem. Severe cases are often secondary to vertebral compression fractures, and OVCFs are common in thoracic and lumbar vertebrae. At the same time, severe chest and back pain can lead to an increase in bed rest time and a great increase in mortality. At present, the treatment of OVCFs is mainly surgical treatment and PVP has become one of the main surgical methods for this disease because of its high reliability and safety (8).

However, after PVP, the adjacent vertebral body may be re-fractured, reducing the overall curative effect (9). Previous studies have found that low bone mineral density, preoperative multiple vertebral fractures, distribution of bone cement (10), noise of anti-osteoporotic drugs (11), increased age, bone cement injection dose, and other factors (12) may lead to adjacent vertebral body re-fracture after PVP.

Our results showed that there was no significant difference in sex, age, and time from the first fracture to the operation between the fracture group and the non-fracture group. There were significant differences in BMI, BMD, bone cement injection, cement leakage, and glucocorticoid use between the fracture group and the non-fracture group. This shows that low BMI, low BMD, large amount of bone cement injection, cement leakage, and the use of glucocorticoid are the risk factors of adjacent vertebral body re-fracture after PVP. In the study of osteoporosis, low BMI is a risk factor for vertebral fracture, and people who are thinner are more likely to have re-fracture (13). On the one hand, weight loss will lead to bone loss, which will exert a mechanical load on bones, and bone mass will increase in order to cope with the mechanical load (14); on the other hand, adipose tissue is one of the main sources of estrogens, especially in post-menopausal women, estrogen inhibits osteoclast activity, increases BMD, promotes bone formation, and reduces the risk of vertebral re-fracture (15). Post-operative re-fracture is closely related to low BMD, but studies (16, 17) have shown that vertebral re-fracture is not an inevitable process of osteoporosis. Bone cement leakage is one of the common complications after PVP; especially, the leakage of bone cement to the intervertebral disc will lead to excessive stress concentration of the intervertebral disc and re-fracture of the adjacent vertebral body (18).

The stability of the spine mainly depends on the vertebral body, muscle and nerve system, and a disorder of any one of them may lead to spinal dysfunction (19). As an important structure to stabilize the spine, the paraspinal muscle is mainly composed of the anterior psoas major muscle and posterior multifidus and erector spinae. There are also common paracrine, endocrine, genetic, mechanical mechanisms, and molecular signal regulation pathways (20). A study (21) found that the smaller the cross-sectional area of the paraspinal muscles, the smaller the tension. When the spine is subjected to an internal or external load, the buffering and stabilizing effect of the paraspinal muscles on the spine will be weakened. Related studies have pointed out that the results of single-segment muscle measurement can predict overall muscle function (22). Our study chose to measure the image data of paraspinal muscles in the median plane of L3/4 intervertebral disc to speculate the effect of paraspinal muscle degeneration on adjacent vertebral re-fractures after PVP.

In previous studies, the CSA of muscle is usually used to represent the number of muscle fibers, and the FIR represents muscle mass (23). Muscle degeneration usually means a decrease in muscle volume and an increase in fat infiltration because muscle satellite cells differentiate into adipocytes under pathological conditions, resulting in the accumulation of intermuscular adipose tissue (24). Intermuscular fat infiltration can lead to a decrease in muscle density (25), and the decrease in paraspinal muscles will reduce BMD (26). Kim et al. (27) found that the area of paraspinal muscles decreased and fat infiltration increased after an osteoporotic vertebral compression fracture. It has also been found that a decline in muscle mass can lead to gait instability (28), and falls are one of the important factors of fractures. A controlled study (29) shows that there is a correlation between bone mineral density and the cross-sectional area of paraspinal muscles, and a large number of previous studies have confirmed that lower bone mineral density increases the risk of vertebral re-fracture after PVP (30–32). The results showed that the cross-sectional area of multifidus and erector spinae in the non-fracture group was larger than that in the fracture group, and the fat infiltration rate of multifidus and erector spinae in the fracture group was significantly higher than that in the non-fracture group (all *p* < 0.05). The degeneration of the posterior group of spinal muscles (multifidus and erector spinae) was a risk factor for re-fracture after PVP.

In the process of paraspinal muscle degeneration, the severity of the left and right sides may be different, which is also one of the important factors of spinal instability (33–35). The deep multifidus and the superficial erector spine muscle play an important role in the stability of the trunk (36). If they degenerate, it will lead to the imbalance of the spine in the sagittal position (37, 38), leading to the forward shift of the whole center of gravity and the compensatory increase of the oblique angle of the pelvis (39). The asymmetric distribution of multifidus is a risk factor for degenerative spondylolisthesis (40). Our study further shows that the asymmetrical distribution of posterior spinal muscles (multifidus and erector spinae) is an independent risk factor for adjacent vertebral re-fracture after PVP. This may be due to the special anatomical position of the multifidus and erector spinae, and the asymmetrical distribution of muscles can aggravate the instability of the spine.

To sum up, the risk of vertebral re-fracture after PVP is high, and the factors affecting vertebral re-fracture after PVP are various. As the most important muscle to stabilize the lumbar spine, the degeneration of the posterior group of muscles will lead to a decrease in spinal stability. Our study proved the effect of degeneration of paraspinal muscles (especially multifidus and erector spinae) on the re-fracture of adjacent vertebrae after PVP treatment of osteoporotic vertebral compression fractures.

Finally, our research also has some inadequacies. The main limitation is that our study only included the data before the first operation, which may be biased; second, this is a retrospective study, and we focused on paraspinal muscles factors based on MRI, the lack of pathological and histological basis; but our study provides a key insight for paraspinal muscles to re-fracture after PVP, which may play a guiding role in the treatment of osteoporotic vertebral compression fractures.

## Author contributions

MC and CY: conceptualization, methodology, software, investigation, formal analysis, and writing—original draft. ZC, YL, and HL: data curation and writing—original draft. YC: visualization and investigation. JC and ZY: conceptualization, funding acquisition, resources, supervision, and writing—review and editing. All authors contributed to the article and approved the submitted version.
